# Plant-derived alginate polysaccharide hydrogels in sport and exercise nutrition: implications for carbohydrate metabolism, gastrointestinal integrity, exercise recovery, and athletic performance

**DOI:** 10.3389/fnut.2026.1774380

**Published:** 2026-04-10

**Authors:** Pengyuan Li, Qingwei Song, Nannan Xu

**Affiliations:** 1 School of Sport and Health, Zhumadian Preschool Education College, Zhumadian, Henan, China; 2 School of Biotechnology and Food Science, Tianjin University of Commerce, Tianjin, China

**Keywords:** carbohydrate hydrogel, endurance performance, exercise recovery, gastrointestinal integrity, sport and exercise nutrition

## Abstract

**Background:**

Alginate is a plant-derived polysaccharide used in sport nutrition for its gel-forming properties. Alginate- and pectin-based carbohydrate hydrogels aim to optimize carbohydrate delivery during endurance exercise by modulating gastric emptying and intestinal absorption. However, evidence on their effects on metabolism, gastrointestinal (GI) tolerance, recovery, and performance remains inconsistent.

**Objective:**

To systematically evaluate the effects of alginate-based carbohydrate hydrogels on metabolic responses, GI tolerance, recovery, and endurance performance in humans.

**Methods:**

PubMed, Scopus, Web of Science, SPORTDiscus, and Google Scholar were searched for randomized controlled trials (2000–2025) comparing alginate- or alginate–pectin–based hydrogels with non-hydrogel carbohydrate or placebo during ≥60-min endurance exercise. Outcomes on metabolism, GI symptoms, recovery, and performance were qualitatively synthesized and visualized via an evidence heatmap.

**Results:**

Nine trials met inclusion criteria. Hydrogels generally enhanced metabolic indicators such as exogenous carbohydrate oxidation, particularly at high ingestion rates. GI tolerance was similar to traditional solutions, with fewer symptoms reported under high-carbohydrate conditions. Performance effects were inconsistent–minor improvements occurred mainly in running, with limited or null effects in cycling and skiing. Preliminary evidence indicates potential attenuation of muscle damage markers and preserved post-exercise amino acid availability.

**Conclusion:**

Alginate-based hydrogels appear to modulate carbohydrate metabolism without consistently improving endurance performance. Their efficacy depends on exercise type, carbohydrate dose, and athlete training status. Further well-controlled trials are needed to define their role in supporting performance and recovery.

## Introduction

Carbohydrate ingestion during prolonged endurance exercise is a cornerstone of sport nutrition, with extensive evidence demonstrating benefits for maintaining blood glucose homeostasis, sustaining high rates of carbohydrate oxidation, delaying fatigue, and supporting endurance performance (1–5). Current guidelines recommend carbohydrate intakes of up to 90 g⋅h^–1^ or higher during prolonged or high-intensity exercise, particularly when multiple transportable carbohydrates such as glucose and fructose are consumed (6–8). However, the practical implementation of these recommendations is often limited by gastrointestinal (GI) symptoms–including nausea, bloating, abdominal discomfort, and diarrhea–that may compromise both performance and adherence to nutritional strategies (1, 9, 10). Improving the efficiency and tolerance of carbohydrate delivery during exercise therefore remains a major focus of applied nutrition research (1). In this context, alginate-based carbohydrate hydrogels have emerged as an innovative, food-technology–driven approach within sport nutrition (9, 11). These formulations typically combine carbohydrates with naturally occurring polysaccharides such as sodium alginate and pectin, which are widely utilized in food systems (12). Under acidic gastric conditions, these compounds are proposed to form a hydrogel matrix that encapsulates carbohydrates, potentially altering gastric physicochemical properties and influencing nutrient delivery to the small intestine (13, 14).

Mechanistically, hydrogel-based carbohydrate strategies may reduce gastric osmolality, modify gastric emptying kinetics, and enhance intestinal carbohydrate availability, thereby supporting higher rates of exogenous carbohydrate oxidation while improving GI comfort (15, 16). Additional proposed benefits include reduced exercise-induced GI symptoms and decreased dental acid exposure due to altered oral carbohydrate release (17–19). Additional putative benefits include reduced exercise-associated GI symptoms and attenuated dental acid exposure due to modified oral carbohydrate availability (17). Such hypotheses position hydrogel formulations at the intersection of food chemistry, gastrointestinal physiology, and human metabolism. Since their introduction to the sports nutrition market, alginate-based hydrogel products have been widely adopted by endurance athletes across disciplines such as running, cycling, and cross-country skiing. Alongside their commercial uptake, an expanding body of research has examined their metabolic, gastrointestinal, recovery-related, and performance effects under controlled laboratory and field conditions (17, 20). Findings remain inconsistent: some studies report enhanced exogenous carbohydrate oxidation, improved GI tolerance, or modest performance gains under specific circumstances, whereas others show no meaningful advantage over traditional carbohydrate solutions when composition and dosage are matched (17, 21–29).

The efficacy of alginate-based hydrogel formulations appears highly context dependent. Factors such as ingestion rate, exercise modality, environmental conditions, athlete training status, and inclusion of other nutrients (e.g., protein or branched-chain amino acids) may all influence outcomes (17, 21–29). Moreover, variability in study design, participant characteristics, and outcome measures complicates interpretation and limits translation into evidence-based recommendations.

Accordingly, the aim of this systematic review is to synthesize current evidence on alginate-based carbohydrate hydrogel formulations in endurance exercise from a human nutrition perspective. Specifically, we evaluate their effects on carbohydrate metabolism, gastrointestinal integrity and tolerance, exercise performance, and recovery outcomes, while highlighting key methodological limitations and future research priorities. By integrating findings from studies published between 2000 and 2025, this review provides an evidence-informed framework to guide researchers and practitioners in applying hydrogel-based carbohydrate strategies within sport and exercise nutrition.

### PRISMA-style methods and study selection

**FIGURE 1:**
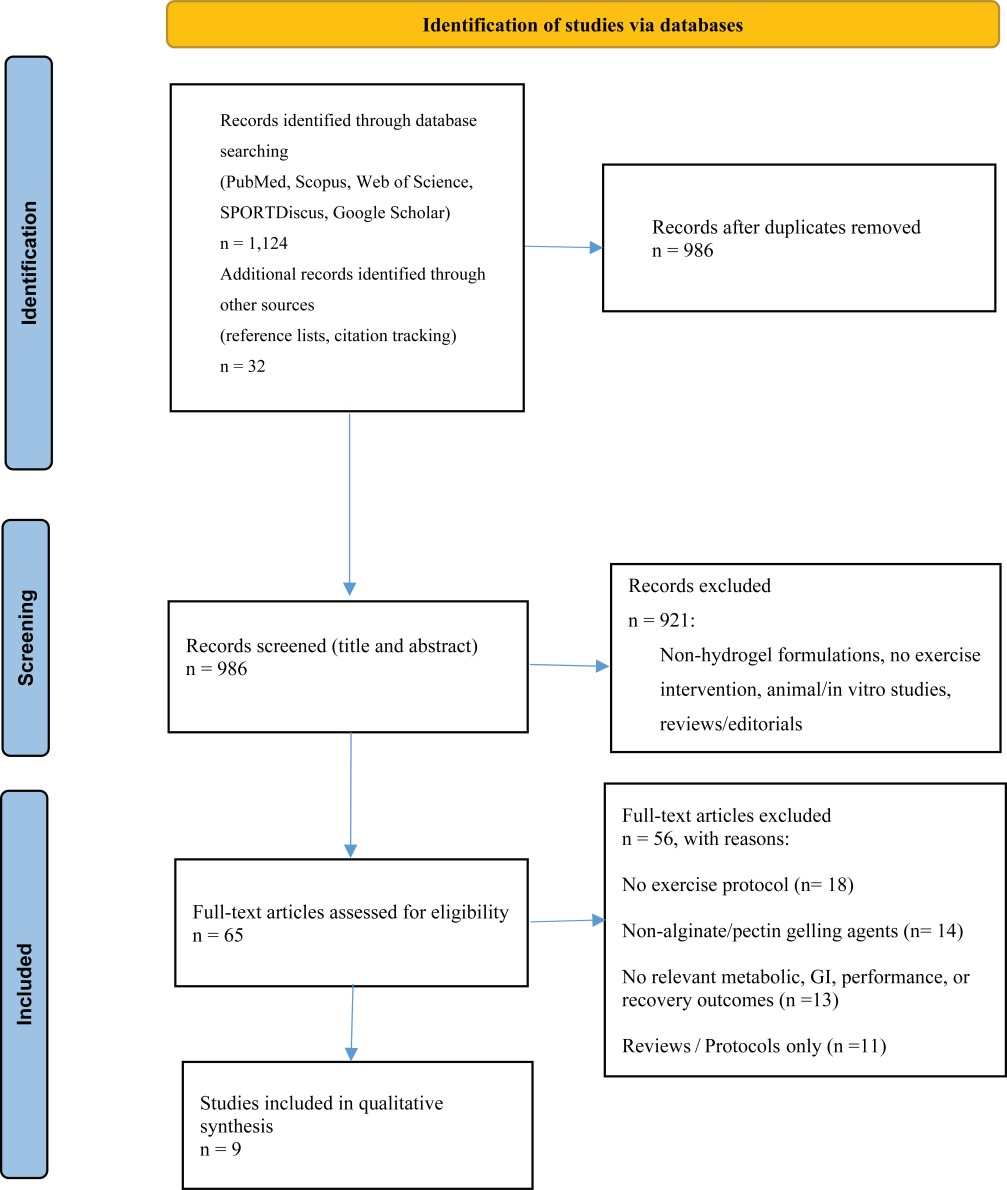
Search strategy flow chart for systematic reviews.

This systematic review was conducted in accordance with the Preferred Reporting Items for Systematic Reviews and Meta-Analyses (PRISMA) guidelines (Figure 1). A comprehensive literature search was performed in PubMed, Scopus, Web of Science, SPORTDiscus, and Google Scholar to identify studies published between January 2000 and March 2025. Search terms were combined using Boolean operators and included: hydrogel, alginate, pectin, carbohydrate, glucose, fructose, exercise, endurance, performance, and oxidation. The reference lists of eligible articles and relevant narrative reviews were also hand-searched to identify additional studies. Reference lists of eligible articles and relevant narrative reviews were also manually screened to identify additional studies.

### Eligibility criteria

Studies were included if they met the following criteria:

1. Employed a randomized controlled or randomized crossover design;

2. Involved human participants (trained or recreational);

3. Compared alginate- or alginate–pectin–based carbohydrate hydrogel formulations with matched non-hydrogel carbohydrate solutions or water/placebo;

4. Included an endurance exercise protocol lasting at least 60 min;

5. Reported at least one outcome related to carbohydrate metabolism (e.g., exogenous carbohydrate oxidation or substrate utilization), gastrointestinal symptoms or integrity, exercise performance or capacity, or recovery-related biomarkers.

Studies qualified for inclusion only if they examined oral hydrogel formulations containing alginate or pectin within exercise settings involving human participants. Exclusion criteria encompassed studies using *in vitro* or animal models, non-alginate/pectin gelling agents, or those without an exercise component. Abstracts, editorials, commentaries, and opinion pieces were also excluded.

### Study selection process

Screening followed a two-stage procedure. First, titles and abstracts retrieved through the database search were independently reviewed by two investigators to assess eligibility. Second, the full texts of potentially relevant studies were examined by the same reviewers. Any discrepancies were resolved through discussion and consensus; when agreement was not immediately achieved, a third reviewer adjudicated. No formal measure of inter-reviewer agreement (e.g., Cohen’s kappa) was calculated because disagreements were rare and resolved through discussion.

### Data extraction and synthesis

From each included study, data were extracted for the following variables: publication year, study design, sample size, participant characteristics (sex and training status), exercise modality and intensity, hydrogel formulation and carbohydrate dosage, and outcomes related to metabolic modulation, gastrointestinal tolerance, performance, and recovery. Given the substantial heterogeneity across hydrogel formulations, exercise protocols, and outcome measures, findings were synthesized qualitatively to provide a comprehensive overview while acknowledging limited potential for direct quantitative comparison. The review protocol was not registered in a public database such as PROSPERO; this has been explicitly noted to ensure transparency.

### Risk of bias and methodological quality

The methodological quality of the included studies was independently assessed by two reviewers using the Cochrane Risk of Bias tool for randomized trials. Evaluated domains included random sequence generation, allocation concealment, blinding of participants and personnel, blinding of outcome assessment, incomplete outcome data, and selective reporting. Discrepancies were resolved through discussion and consensus. A summary of the risk-of-bias assessment for each study is presented in Table 1, supporting the overall reliability of the evidence base. Overall, the studies were judged to have moderate-to-high methodological quality. All trials adequately reported random sequence generation, resulting in a low risk of bias for this domain. Allocation concealment was clearly described in most cases, although two studies (22, 27) were rated as unclear due to insufficient procedural detail. Blinding of participants, study personnel, and outcome assessors was generally implemented and rated as low risk across studies. Attrition rates were minimal, and incomplete outcome data were appropriately addressed, yielding low risk in this domain. No evidence of selective reporting was observed; reported outcomes were consistent with the stated objectives and methods.

**TABLE 1:** Risk of bias assessment of included studies.

References	Random sequence generation	Allocation concealment	Blinding (participants and personnel)	Blinding (outcome assessment)	Incomplete outcome data	Selective reporting	Overall risk
Nielsen et al. (21)	Low	Low	Low	Low	Low	Low	Low
Nielsen et al. (17)	Low	Low	Low	Low	Low	Low	Low
Rowe et al. (24)	Low	Low	Low	Low	Low	Low	Low
Sutehall et al. (22)	Low	Unclear	Low	Low	Low	Low	Low
Flood et al. (23)	Low	Low	Low	Low	Low	Low	Low
McCubbin et al. (25)	Low	Low	Low	Low	Low	Low	Low
Pettersson et al. (28)	Low	Low	Low	Low	Low	Low	Low
Pettersson et al. (26)	Low	Low	Low	Low	Low	Low	Low
Dean et al. (27)	Low	Unclear	Low	Low	Low	Low	Low

Despite these strengths, several methodological limitations were evident. Sample sizes were typically small, with most trials including fewer than 15 participants, limiting statistical power and generalizability. Study populations were predominantly male, with few investigations exploring sex-specific responses. Considerable variability in exercise modality, duration, intensity, and nutritional protocols further contributed to heterogeneity, highlighting the need for greater standardization in experimental designs. Although the overall risk of bias was low to moderate, these limitations emphasize the necessity for larger, more diverse, and methodologically rigorous trials to better elucidate the effects of alginate- and pectin-based carbohydrate hydrogel formulations on endurance performance, thereby guiding future research efforts in this field.

### Formulation and carbohydrate delivery characteristics

The included studies examined a range of alginate- and pectin-based carbohydrate hydrogel formulations, differing in carbohydrate type, dose, hydrogel matrix composition, and timing of delivery (Table 2). This diversity highlights multiple formulation strategies aimed at optimizing exercise performance and recovery. Hydrogel matrices typically combined sodium alginate with pectin in varying proportions, designed to form gels under the acidic conditions of the stomach (30). This gel formation is hypothesized to modulate gastric emptying and enhance carbohydrate availability during exercise (31), reinforcing its potential utility for sports nutrition and performance enhancement. Reported carbohydrate doses ranged from 0.8 g⋅kg^–1^⋅h^–1^ during recovery to 180 g⋅h^–1^ during exercise, encompassing both typical and supra-physiological intake scenarios (Table 2). Delivery strategies–intermittent, bolus, or post-exercise ingestion–illustrate practical options for tailoring carbohydrate intake to specific performance or recovery objectives.

**TABLE 2:** Formulation and carbohydrate delivery characteristics of hydrogel-based interventions included in the review.

References	Formulation	CHO type and ratio	CHO dose	Hydrogel matrix	Delivery timing
Nielsen et al. (21)	ALG-CHO + PRO	Maltodextrin + plant protein (2:1)	0.8 g CHO⋅kg^–1^⋅h^–1^	Sodium alginate	Recovery
Nielsen et al. (17)	ALG-CP	Maltodextrin + fructose + BCAA	∼90 g⋅h^–1^	Alginate	During exercise
Rowe et al. (24)	CHO hydrogel	Glucose:fructose (2:1)	90 g⋅h^–1^	Alginate + pectin	During exercise
Sutehall et al. (22)	ENCAP/HiENCAP	Maltodextrin:fructose (1:0.7)	70–180 g⋅h^–1^	Alginate + pectin	During exercise
Flood et al. (23)	MAL + FRU + PEC + ALG	Maltodextrin + fructose	90 g⋅h^–1^	Alginate + pectin	During exercise
McCubbin et al. (25)	CES-HGel	Maltodextrin + fructose	90 g⋅h^–1^	Alginate + pectin	During exercise
Pettersson et al. (28)	MD + FRU hydrogel	Maltodextrin + fructose	95 g⋅h^–1^	Alginate	During exercise
Pettersson et al. (26)	CHO-HG	Maltodextrin:fructose (1:0.8)	2.2 g⋅min^1^	Alginate	During exercise
Dean et al. (27)	GF-Gel	Glucose + fructose	45 g bolus	Commercial hydrogel	Pre/intermittent

Collectively, the studies provide a comprehensive overview of current Alginate content hydrogel formulations, highlighting variability in carbohydrate composition, matrix architecture, dosing, and timing–factors that may influence metabolic responses, gastrointestinal tolerance, performance outcomes, and post-exercise recovery. Clarifying how each variable affects these outcomes will guide the design of more targeted and mechanistically informative investigations in future research.

## Discussion

This systematic review evaluated the effects of alginate- and pectin-based carbohydrate hydrogel formulations (ALG-CP) on metabolic responses, gastrointestinal integrity, exercise recovery, and athletic performance across controlled trials published between 2000 and 2025. The consolidated findings (Table 3) indicate substantial methodological and outcome heterogeneity, suggesting that the ergogenic efficacy of hydrogel formulations is context-dependent rather than universally beneficial.

**TABLE 3:** Summary of participant characteristics, hydrogel formulations, and key outcomes across included studies.

References	Formulation	Sample size/participant characteristics	Exercise recovery	GI integrity/ tolerance	Metabolic modulation	Performance outcomes
Nielsen et al. (21)	ALG:alginate-encapsulated plant-based CHO + protein (0.8 g CHO/kg/hr + 0.4 g PRO/kg/hr) vs. CON (1.2 g CHO/kg/hr)	*n* = 14, trained male cyclists	5 h recovery; plasma BCAAs higher with ALG	Saliva pH similar (no adverse GI effects)	ALG ↑ glucagon, free fatty acids and glycerol; CON ↑ insulin/glucose	TTE performance similar (*p* = 0.13) despite ALG 1/3 less CHO
Nielsen et al. (17)	ALG-CP (alginate CHO + BCAA) vs. ALG-C (alginate CHO) vs. CON (non-hydrogel CHO)	*n* = 10, trained male cyclists/triathletes	Not measured post-exercise; lower myoglobin (reduced muscle damage) with ALG-CP	GI symptoms minimal; hydrogel tolerated	ALG-CP ↑ early insulin; glucagon higher pre-TTE	TTE longer with ALG-CP vs. ALG-C and CON (≈ + 29%) (NS) with lower HR
Sutehall et al. (22)	Alginate + pectin CHO drink (ENCAP); HiENCAP (180 g/h); NORM CHO (70 g/h)	*n* = 8, well-trained runners	Not measured	GI discomfort similar to control and water	Peak ExGluc higher for HiENCAP (180 g/h)	No performance test
Flood et al. (23)	MAL + FRU + PEC + ALG vs. MAL + FRU vs. water	*n* = 14, recreational cyclists	Not measured	Both CHO drinks ↓ I-FABP and GI permeability vs. water; hydrogel no extra benefit	No metabolic differences between CHO drinks	15-min TT unchanged
Rowe et al. (24)	90 g/h hydrogel (alginate + pectin, 2:1 glucose:fructose)	*n* = 11, trained male runners	Not measured	Hydrogel ↓ GI symptoms vs. non-hydrogel; similar to placebo	Hydrogel ↑ ExCHO; ↓ endogenous CHO	Hydrogel 5-km TT faster vs. non-hydrogel and placebo
McCubbin et al. (25)	CES-HGel (alginate + pectin CES) vs. CES-Std	*n* = 9, trained male endurance runners	Not measured	No difference in GI symptoms; high incidence both	No difference in blood glucose or substrate oxidation	TTE unchanged (HGel 722 vs. Std 756 s)
Pettersson et al. (26)	18% CHO hydrogel (1:0.8 maltodextrin:fructose, gelling polysaccharides; 13C-enriched; ∼132 g CHO/h)	*n* = 12, endurance cyclists; VO_2_max ∼65.6 mL/kg/min	Not measured	Dental biofilm pH less acidic with MD + FRU	Exogenous CHO oxidation highest with MD + FRU	No performance test
Dean et al. (27)	GF-Bar (glucose–fructose bar) vs. GF-Gel (glucose–fructose hydrogel) vs. MD-Gel (maltodextrin gel) – each providing 45 g CHO	*n* = 12, Tier 2 trained athletes	Not measured	GI discomfort similar across formats	GF-Bar > GF-Gel > MD-Gel for CHO oxidation	Sprint performance similar
Pettersson et al. (28)	MD + FRU hydrogel (maltodextrin + fructose, 14%) vs. MD + SUC (maltodextrin + sucrose, 14%) vs. AP (amylopectin starch, 14%) each providing 95 g CHO/h	*n* = 12 (6 F/6 M) elite XC skiers	Not measured	Well tolerated; no severe GI symptoms	Peak ExCHO 1.33 g/min; ↑ total CHO oxidation, ↓ fat oxidation	No TT performance benefit

↑Increase, ↓decrease.

### Metabolic modulation

**FIGURE 2:**
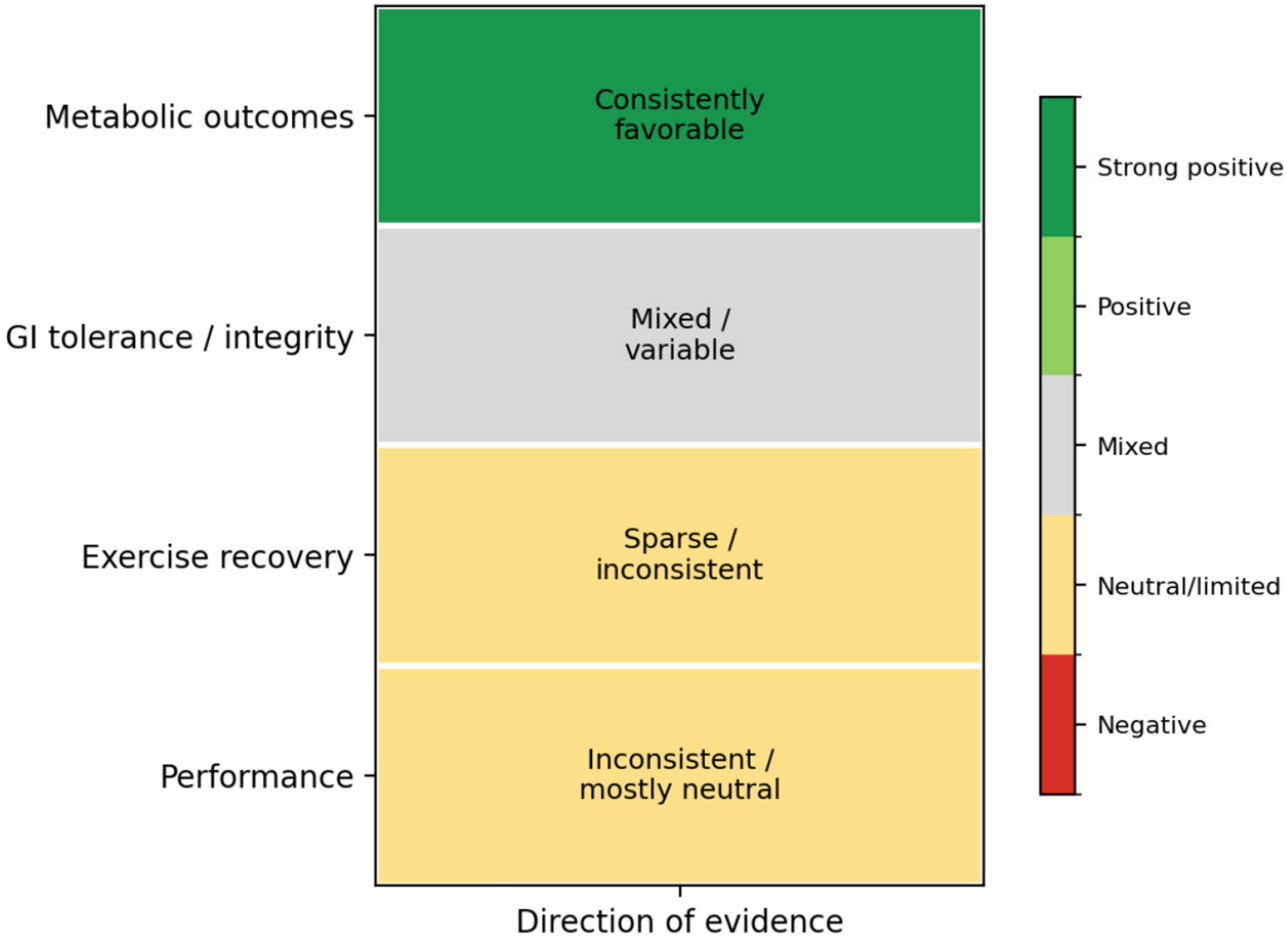
Integrated evidence heatmap summarizing the reported effects of alginate-pectin carbohydrate hydrogel formulations across metabolic, gastrointestinal, performance, and recovery outcomes. Shades indicate the direction and consistency of reported effects within each outcome domain. The visualization highlights context-dependent patterns across heterogeneous study protocols, illustrating that while hydrogel ingestion is consistently associated with improved carbohydrate oxidation, its influence on gastrointestinal tolerance, performance, and recovery outcomes remains variable.

Across the reviewed literature, metabolic outcomes emerged as the most consistently affected domain, underscoring their relevance for researchers and practitioners in sports nutrition and metabolism. At moderate carbohydrate ingestion rates (≤70 g⋅h^–1^), hydrogel formulations generally do not enhance exogenous carbohydrate oxidation (ExCHO) beyond levels achieved with conventional multiple-transportable carbohydrate solutions (22, 25, 29). These findings indicate that intestinal transporter saturation, rather than gastric emptying or encapsulation efficiency, is the principal limiting factor under these conditions. Conversely, at very high ingestion rates (≥90–180 g⋅h^–1^), alginate-based hydrogels consistently support exceptionally high ExCHO oxidation rates, often exceeding 1.1–1.3 g⋅min^1^ (22, 26, 28). These studies dominate the “positive” metabolic outcomes in the heatmap (Figure 2) and reinforce the mechanistic premise that hydrogel formation may accelerate gastric emptying while delaying carbohydrate release until intestinal transit, strengthening confidence in these findings among sports scientists and nutritionists. Notably, Nielsen et al. (21) extended metabolic investigation beyond substrate oxidation by examining recovery-phase endocrine responses. Alginate-encapsulated carbohydrate–protein supplementation elicited lower insulin and glucose concentrations, accompanied by higher plasma amino acids, glucagon, free fatty acids, and glycerol compared with an isocaloric carbohydrate-only condition. Although these alterations did not translate into improved time-to-exhaustion performance, they provide valuable insight into metabolic partitioning during recovery–potentially relevant to muscle repair and metabolic flexibility–and inspire further targeted research.

### Gastrointestinal integrity and tolerance

Gastrointestinal outcomes show mixed but generally neutral-to-positive effects (Figure 2). Subjective GI symptoms are often reduced or unchanged following hydrogel ingestion compared with standard carbohydrate solutions–particularly during running-based protocols, where mechanical and splanchnic stress are elevated (24, 25). Rowe et al. (24) presented one of the clearest examples, reporting significantly lower GI symptom scores with hydrogel ingestion at 90 g⋅h^–1^ compared with a non-hydrogel glucose–fructose solution. The clinical relevance of these symptom reductions for athlete performance and comfort, however, remains uncertain and warrants further investigation to guide practical recommendations. When GI integrity was assessed using objective biomarkers, evidence supporting hydrogel superiority was limited. This finding reinforces scientific rigor by indicating that current data do not overstate potential benefits. Flood et al. (23) found that carbohydrate ingestion attenuated exercise-induced increases in intestinal fatty acid-binding protein (i-FABP) and gut permeability relative to water; however, pectin–alginate hydrogels conferred no additional protection compared with matched carbohydrate beverages. These results suggest that carbohydrate availability itself, rather than encapsulation, is the primary factor in maintaining GI barrier integrity during prolonged exercise, especially in hot and humid conditions.

A less frequently examined outcome related to gastrointestinal health is oral health. Pettersson et al. (28) observed that hydrogel formulations produced a smaller decline in dental biofilm pH compared with acidic sports drinks, indicating a potential advantage for athletes with high training volumes and frequent carbohydrate exposure. This highlights a broader practical value of hydrogel formulations beyond GI symptom reduction, supporting their role in promoting overall athlete health and recovery.

### Athletic performance

Performance outcomes across hydrogel-based carbohydrate studies are highly variable, representing the most inconsistent domain within the current evidence base (Table 3). The majority of randomized controlled and crossover trials reported no statistically significant improvements in conventional performance metrics–such as time-to-exhaustion or time-trial completion–when alginate- or pectin-based hydrogels were compared with carbohydrate- and energy-matched non-hydrogel solutions (25, 26, 29). These null findings occur most often in cycling protocols at moderate relative intensities, where gastrointestinal strain is comparatively lower and carbohydrate delivery demands may not exceed the absorptive capacity of multiple-transportable carbohydrate formulations. In contrast, several studies suggest that hydrogel formulations may offer context-specific performance advantages, particularly under exercise conditions characterized by greater GI perturbation or higher absolute carbohydrate flux (25, 29). Rowe et al. (24) reported an approximately 2% improvement in 5-km running time-trial performance following ingestion of a glucose–fructose hydrogel compared with a standard non-hydrogel carbohydrate solution. This improvement was accompanied by significantly higher exogenous carbohydrate oxidation and lower GI symptom scores, supporting the premise that enhanced GI tolerance and carbohydrate availability may underpin performance gains in running-based endurance tasks. Because running imposes greater mechanical stress on the gastrointestinal system than cycling, these results may reflect a larger relative advantage of encapsulated carbohydrate delivery in such modalities.

Similarly, Nielsen et al. (17) observed a longer time to exhaustion during cycling and lower heart rates during prolonged exercise. They reduced post-exercise myoglobin concentrations following the ingestion of an alginate-encapsulated carbohydrate-BCAA supplement. Although some performance-related outcomes did not reach conventional thresholds for statistical significance, the magnitude and direction of these effects suggest potential practical relevance, particularly for competitive athletes, where small physiological advantages may translate into meaningful performance outcomes. The concomitant reduction in myoglobin further raises the possibility that improved muscle integrity or attenuated fatigue development may indirectly support performance maintenance. Collectively, these findings indicate that the performance benefits of hydrogels are not universal and should not be expected under all exercise conditions. Benefits appear most likely when carbohydrate intake rates are high (≥90 g⋅h^–1^), gastrointestinal stress is elevated, or when metabolic and mechanical demands converge–such as during prolonged running, high-intensity endurance exercise, or scenarios requiring sustained carbohydrate availability late in exercise. Conversely, in moderate-intensity cycling or controlled laboratory protocols with well-tolerated carbohydrate solutions, hydrogels appear to offer only marginal or negligible ergogenic advantages beyond those provided by conventional multiple-transportable carbohydrate formulations.

### Exercise recovery

Exercise recovery represents the least explored outcome domain in the current hydrogel literature (Figure 2), with only a limited number of studies specifically designed to investigate post-exercise physiological responses rather than in-exercise metabolism or performance. The most direct evidence to date comes from two complementary studies by Nielsen et al. (17, 21), which collectively provide initial insight into the recovery-related effects of alginate-encapsulated carbohydrate–protein and carbohydrate–BCAA formulations. In these randomized crossover trials, ingestion of alginate-based hydrogels during or following prolonged cycling was associated with sustained elevations in circulating amino acids during recovery and significantly lower post-exercise myoglobin concentrations compared with carbohydrate-only or non-hydrogel comparators. These findings suggest that hydrogel delivery may enhance amino acid availability and attenuate markers of exercise-induced muscle damage, potentially through altered gastric emptying, improved nutrient release kinetics, or enhanced intestinal tolerance under physiological stress.

Importantly, these recovery-related effects were observed despite minimal or non-significant improvements in subsequent time-to-exhaustion performance, indicating that biochemical markers of recovery may be more sensitive to hydrogel formulation effects than short-term performance outcomes. This dissociation highlights a critical gap in the literature: while blood-based biomarkers such as myoglobin, insulin, and plasma amino acids provide valuable mechanistic insight, they do not necessarily translate into functional recovery. Notably absent across studies are validated measures of neuromuscular recovery, including strength restoration, repeated-bout performance capacity, muscle soreness, and perceptual recovery indices assessed over multiple days. Moreover, recovery outcomes have largely been evaluated within tightly controlled laboratory settings, typically over short post-exercise windows (<6 h). This narrow temporal focus limits ecological validity for real-world training and competitive contexts. The potential benefit of hydrogel formulations for recovery may therefore be most relevant in scenarios involving high carbohydrate demands combined with protein or BCAA provision, such as stage racing, tournament play, or intensive training blocks. However, these applications remain speculative in the absence of longitudinal, multi-day intervention studies. Future research should extend beyond acute biochemical responses, employing repeated-exercise models, delayed recovery assessments (24–72 h), and performance-relevant endpoints. Additionally, direct comparisons of hydrogel-based recovery strategies with established carbohydrate–protein solutions are needed to determine whether encapsulation provides unique benefits beyond nutrient composition alone.

### Integrated interpretation

The consolidated evidence heatmap (Figure 2) indicates that alginate- and pectin-based carbohydrate hydrogel formulations are most consistently associated with favorable metabolic outcomes, show variable associations with gastrointestinal tolerance, and display inconsistent links to performance or recovery benefits. Notably, no study has reported adverse metabolic or gastrointestinal effects attributable to hydrogel ingestion, suggesting that these formulations are nutritionally safe and metabolically comparable to conventional carbohydrate solutions. Collectively, the current evidence supports the interpretation that hydrogel-based carbohydrate strategies function as context-specific nutritional tools, rather than as universally superior alternatives to standard carbohydrate delivery methods. Their principal utility appears to lie in facilitating very high carbohydrate intakes under conditions of elevated gastrointestinal and metabolic stress, rather than in providing inherent ergogenic benefits across all endurance exercise contexts. A detailed justification for the exclusion of full-text records during the systematic screening process is provided in Supplementary Table S1 (32–38).

### Practical implications

From an applied sports nutrition perspective, alginate-based carbohydrate hydrogel formulations should be regarded as a targeted tool rather than a universally superior strategy for carbohydrate delivery. This distinction reinforces the importance of individualized approaches for sports nutrition professionals and athletes. Hydrogel formulations appear particularly beneficial for athletes seeking to achieve very high carbohydrate intake rates (typically > 90 g⋅h^–1^). Recognizing this potential can help nutritionists and coaches optimize fueling strategies during prolonged endurance events, enabling athletes to maintain performance while minimizing gastrointestinal discomfort. Endurance running and other exercise modalities characterized by elevated gastrointestinal stress may particularly benefit from hydrogel use, as these formulations can reduce symptoms and support consistent fueling, thereby helping sports scientists and coaches enhance athlete management during competition or extended training sessions.

Hydrogels may also provide value in situations where oral health is a concern. Their lower beverage acidity and encapsulated carbohydrate delivery have been linked to smaller reductions in dental biofilm pH, potentially reducing the risk of dental erosion during repeated carbohydrate exposure across long-duration exercise. Additionally, emerging evidence suggests that hydrogel-based formulations may support exercise recovery, particularly when carbohydrates are co-delivered with protein or branched-chain amino acids (BCAAs). Such combinations can help sustain circulating amino acid availability, attenuate markers of muscle damage, and facilitate recovery processes following prolonged or exhaustive exercise. However, practical factors such as cost, preparation complexity, and athlete preference should be considered when determining their suitability for specific training or competition contexts. Conversely, for moderate-intensity exercise or scenarios where carbohydrate intake remains within standard recommendations (≤60–90 g⋅h^–1^), hydrogel formulations appear to offer no substantial advantage over conventional multiple-transportable carbohydrate solutions. In such cases, simpler carbohydrate beverages remain an effective and practical choice for most athletes.

## Strengths and limitations

### Strengths

This systematic review has several notable strengths. First, the study was conducted in accordance with PRISMA guidelines and employed a transparent, predefined search strategy across multiple databases, thereby enhancing methodological rigor and reproducibility. Only randomized controlled and randomized crossover trials involving human participants were included, ensuring a high level of internal validity and direct relevance to applied sport and exercise nutrition. Second, the review adopts a nutrition-focused perspective by examining alginate- and pectin-based hydrogels as food-grade carbohydrate delivery systems rather than as isolated ergogenic aids. This approach enables the integrated interpretation of metabolic, gastrointestinal, performance, and recovery-related outcomes, reflecting the multifaceted role of carbohydrate ingestion during endurance exercise. Third, outcomes were evaluated across multiple physiological domains, including exogenous carbohydrate oxidation, gastrointestinal tolerance and integrity, performance capacity, and post-exercise recovery markers. The inclusion of recovery and oral health outcomes, which are rarely addressed in this literature, provides additional mechanistic and practical insight. Finally, the qualitative synthesis and evidence heatmap enable clear visualization of context-dependent effects across heterogeneous study designs.

### Limitations

Several limitations of the current evidence base should be acknowledged. Most included studies were characterized by small sample sizes, typically involving fewer than 15 participants, which limits statistical power and increases the risk of type II error. Study populations were predominantly male, which restricts the generalizability of findings to female athletes, who may differ in gastrointestinal function, substrate utilization, and hormonal responses during exercise.

The underrepresentation of female participants warrants more detailed consideration. Sex-specific physiological factors, including differences in gastric emptying rates, intestinal permeability, and substrate metabolism, may meaningfully influence the absorption and tolerance of carbohydrate hydrogels. Moreover, hormonal fluctuations across the menstrual cycle, particularly variations in estrogen and progesterone, can alter gastrointestinal motility, fuel selection, and perceived exertion. These factors could modify both the metabolic and gastrointestinal responses to alginate- or pectin-based carbohydrate ingestion. Consequently, the predominance of male participants limits the external validity of the current evidence base, underscoring the need for future trials to incorporate sex-balanced recruitment, menstrual phase tracking, and dedicated analyses of female-specific responses. Substantial heterogeneity was observed across studies in terms of exercise modality, duration, intensity, environmental conditions, carbohydrate dose, and hydrogel formulation. Outcome measures also varied widely, encompassing metabolic tracer-derived oxidation rates, subjective gastrointestinal symptom scores, and different performance tests. This heterogeneity precluded formal meta-analysis and limited direct quantitative comparisons between studies.

In addition, many trials recruited recreationally trained or sub-elite participants, with relatively few studies involving elite endurance athletes operating at very high absolute workloads. As a result, the contexts in which hydrogel-based carbohydrate strategies are hypothesized to offer the most significant benefit, such as prolonged competition with extreme carbohydrate intake demands, remain underrepresented. Recovery-related outcomes were also sparsely investigated and were often limited to short post-exercise time frames, relying primarily on biochemical markers rather than functional measures of recovery. Recovery-related outcomes are another area of limitation. Most studies employed only short-term biochemical markers, such as muscle glycogen content, plasma cytokines, or creatine kinase activity, to infer recovery, providing limited insight into real-world, and functional restoration of performance. Functional recovery outcomes, such as subsequent exercise capacity, time-trial performance, or perceived readiness, were rarely assessed. Accordingly, the ecological validity of existing recovery data remains low. Future research should incorporate multi-day, integrative designs that simultaneously evaluate biochemical, perceptual, and performance-based measures to better understand how hydrogel ingestion influences recovery in practical athletic contexts. Finally, inter-individual variability, sex-specific responses, and potential adaptations to repeated hydrogel use have not been systematically examined. These gaps highlight the need for larger, more diverse, and methodologically standardized trials to define better the role of alginate-based carbohydrate hydrogels in endurance exercise nutrition. A further consideration concerns potential commercial and industry-related bias. Alginate-based carbohydrate hydrogel formulations are increasingly commercialized as proprietary products, and several included trials relied on manufacturer-supplied supplements or partial industry sponsorship. Although most authors declared no conflicts of interest, commercial involvement may subtly affect study design, comparator choice, or interpretation, favoring positive outcomes. Publication bias may also amplify beneficial findings in industry-supported research. Consequently, independent replication under investigator-initiated conditions, transparent funding disclosure, preregistration, and open data availability are recommended to safeguard neutrality and maintain scientific integrity in this evolving research domain.

## Conclusion

This systematic review synthesized evidence from controlled human trials evaluating alginate- and pectin-based carbohydrate hydrogel formulations in endurance exercise, with a focus on carbohydrate metabolism, gastrointestinal tolerance and integrity, exercise performance, and recovery-related outcomes. The consolidated evidence indicates that hydrogel formulations are most consistently associated with favorable metabolic responses, particularly under conditions of very high carbohydrate intake, where exogenous carbohydrate oxidation rates exceed those typically achieved with conventional multiple-transportable carbohydrate solutions. In contrast, the effects on gastrointestinal tolerance, athletic performance, and exercise recovery are more variable and highly context-dependent. Subjective gastrointestinal symptoms are often reduced or unchanged with hydrogel ingestion, especially in running-based protocols characterized by elevated mechanical and splanchnic stress. However, objective markers of gastrointestinal integrity do not consistently demonstrate additional protection beyond that provided by carbohydrate availability alone. Performance benefits are not universal and appear most likely to emerge when carbohydrate intake demands are extreme, gastrointestinal stress is high, or when metabolic and mechanical challenges converge late in prolonged endurance exercise. Evidence relating to post-exercise recovery remains limited but suggests that alginate-based hydrogel formulations may influence nutrient availability and attenuate selected biomarkers of muscle damage when combined with protein or branched-chain amino acids. Importantly, these biochemical responses do not consistently translate into short-term performance improvements, underscoring the need for future studies to incorporate functional and ecologically valid recovery endpoints. Taken together, the current literature supports the view that alginate-based carbohydrate hydrogels represent a context-specific nutritional strategy, rather than a universally superior alternative to conventional carbohydrate formulations. Their primary utility lies in facilitating the delivery of very high carbohydrates under conditions of elevated metabolic and gastrointestinal demand. Further well-controlled, nutrition-focused trials are needed to clarify their role in supporting performance and recovery across diverse endurance exercise settings.

## Data Availability

The original contributions presented in this study are included in this article/Supplementary material, further inquiries can be directed to the corresponding author.
