# Supplementary material for: Plant-derived alginate polysaccharide hydrogels in sport and exercise nutrition: implications for carbohydrate metabolism, gastrointestinal integrity, exercise recovery, and athletic performance
Source: Front Nutr. 2026 Apr 10;13:1774380. doi: 10.3389/fnut.2026.1774380 (PMC13106452; doi:10.3389/fnut.2026.1774380)
Supplement: Supplementary file 1 [file Table_1.docx]

**Supplementary Table S1.** Rationale for full‑text exclusions during systematic screening

| Exclusion Category | Approximate No. of Articles (n = 55) | Representative Examples | Rationale for Exclusion |
| --- | --- | --- | --- |
| No exercise protocol | 18 | Carboxymethyl chitosan and sodium alginate oxide pH-sensitive dual-release hydrogel for diabetes wound healing: The combination of astilbin liposomes and diclofenac sodium (32); How the combination of alginate and chitosan can fabricate a hydrogel with favorable properties for wound healing (33) | Studies did not include a controlled exercise or physical activity intervention. These were excluded because the systematic review focused on alginate supplementation effects during or following exercise protocols. |
| Non‑alginate/pectin gelling agents | 14 | Bioactive scaffolds based on elastin‑like materials for wound healing (34) | The intervention material differed substantively from the alginate or pectin matrix (e.g., xanthan, carrageenan, guar gum). Such formulations do not fit the inclusion criteria targeting alginate‑based gels. |
| No relevant metabolic, GI, performance, or recovery outcomes | 13 | Hydrogels and Wound Healing: Current and Future Prospects (35); Alginate dressings for treating pressure ulcers (36) | Studies reported unrelated endpoints (e.g., psychological measures or unrelated biomarkers). These did not contribute to the core themes of exercise metabolism, gastrointestinal tolerance, or recovery. |
| Reviews / Protocols only | 10 | The efficient role of sodium alginate‑based biodegradable dressings for skin wound healing application: a systematic review (37);  Chitosan, alginate, hyaluronic acid and other novel multifunctional hydrogel dressings for wound healing: A review (38) | Excluded because they lack full methodological detail or peer‑reviewed outcome data. |
